# Particle Radiation-Induced Nontargeted Effects in Bone-Marrow-Derived Endothelial Progenitor Cells

**DOI:** 10.1155/2015/496512

**Published:** 2015-05-05

**Authors:** Sharath P. Sasi, Daniel Park, Sujatha Muralidharan, Justin Wage, Albert Kiladjian, Jillian Onufrak, Heiko Enderling, Xinhua Yan, David A. Goukassian

**Affiliations:** ^1^Cardiovascular Research Center, GeneSys Research Institute, Boston, MA 02135, USA; ^2^Whitaker Cardiovascular Institute, Boston University School of Medicine, Boston, MA 02118, USA; ^3^Department of Integrated Mathematical Oncology, H. Lee Moffitt Cancer Center and Research Institute, Tampa, FL 33612, USA; ^4^Tufts University School of Medicine, Boston, MA 02118, USA

## Abstract

Bone-marrow- (BM-) derived endothelial progenitor cells (EPCs) are critical for endothelial cell maintenance and repair. During future space exploration missions astronauts will be exposed to space irradiation (IR) composed of a spectrum of low-fluence protons (^1^H) and high charge and energy (HZE) nuclei (e.g., iron-^56^Fe) for extended time. How the space-type IR affects BM-EPCs is limited. In media transfer experiments *in vitro* we studied nontargeted effects induced by ^1^H- and ^56^Fe-IR conditioned medium (CM), which showed significant increase in the number of p-H2AX foci in nonirradiated EPCs between 2 and 24 h. A 2–15-fold increase in the levels of various cytokines and chemokines was observed in both types of IR-CM at 24 h. *Ex vivo* analysis of BM-EPCs from single, low-dose, full-body ^1^H- and ^56^Fe-IR mice demonstrated a cyclical (early 5–24 h and delayed 28 days) increase in apoptosis. This early increase in BM-EPC apoptosis may be the effect of direct IR exposure, whereas late increase in apoptosis could be a result of nontargeted effects (NTE) in the cells that were not traversed by IR directly. Identifying the role of specific cytokines responsible for IR-induced NTE and inhibiting such NTE may prevent long-term and cyclical loss of stem and progenitors cells in the BM milieu.

## 1. Introduction

Long lasting, up to 2 years, ionizing radiation- (IR-) induced chromosomal instability had been reported* in vivo* in the bone marrow (BM) after full body exposure to X-rays or neutrons [1, 2]. In addition, it has been shown that after space flights the number of myeloid and lymphoid BM-derived stem and progenitor cells were reduced to just one-half of their normal population [3]. In spite of these reports there is significant gap in assessing the effects of low-dose full body IR on the survival and function of BM stem and progenitor cells, including BM-derived endothelial progenitor cells (BM-EPCs). These earlier findings suggest that the number of EPCs may be similarly reduced in the normal BM-EPC population during and after space flights. Additionally, IR-induced DNA damage in BM may affect significantly the number and function of BM-EPCs. Subsequently reduced number and function in EPCs and other BM stem and progenitor cell populations may affect adversely cardiac homeostasis during normal aging, as well as the repair and regeneration processes after cardiac injury.

Radiobiological bystander responses (RBR) are the phenomena in which nonirradiated (Non-IR) cells exhibit responses similar to effects manifested by IR cells as a result of signals received from either nearby or distant IR cells. Radiobiological bystander responses of IR on a variety of primary and tumor cells have been well-documented* in vitro* [4–10]. RBR-mediated effects can be attributed to events initiated near the Non-IR cell surface that in turn activates and integrates various intracellular signaling pathways that are regulated by RBR [11]. It is important to clarify here that the ability to induce RBR [7] and the ability to receive the IR-induced RBR signaling is cell-, cytokine-, and chemokine-specific [4]. Further, specific ligand-receptor interactions on Non-IR cells may play key role in the propagation of RBR [4, 12, 13] in the remote site from the original site of IR exposure cells and tissues, including cells in the BM milieu.

Our focus on BM-EPCs stems from considerable body of evidence regarding the role of EPCs in repair and regeneration and postnatal angiogenesis (neovascularization) processes after ischemic injury. In various animal models [14–17] and human clinical trials [18–21] our laboratory and others have shown that transplantation of BM cells and BM-EPCs leads to migration and homing of these cells to the areas of damage, where EPCs contribute to the processes of neovascularization leading to the development of collateral vessels, which then contribute to the recovery of blood flow in the damaged tissue such as the heart [22–26], hind limb [27–29], bone [30–33], liver [34–36], and brain and spinal cord [37–41]. Consequently a decrease in the total number of BM-EPCs or their dysfunction could contribute to the pathogenesis of ischemic and/or peripheral vascular diseases. This could also have negative impact on the recovery after tissue injury, as well as negatively affect the maintenance of normal vascular homeostasis in the organs and tissue in general. We therefore tested whether BM-derived EPCs may exhibit radiobiological bystander responses* in vitro* and determined the effect of low-dose full-body particle IR on the survival of BM-derived EPCs* in vivo*.

## 2. Material and Methods

### 2.1. Animal Models

To determine low-dose full-body proton (^1^H)-IR and iron (^56^Fe)-IR induced effects on survival of BM-derived EPCs, adult 8–10 months old male C57Bl/6NTac mice were shipped directly from Taconic (Hudson, NY) to Brookhaven National Laboratory (BNL) to be irradiated at NASA Space Radiation Laboratory (NSRL). Mice were kept in the temperature- and light-controlled environment and handled in accordance with IACUC guidelines and protocols approved by GeneSys Research Institute (GRI) and BNL.

### 2.2. Radiation and Dosimetry

Full-body low-dose space-type IR experiments for low linear energy transfer (LET) ^1^H-IR and high-LET ^56^Fe-IR exposures were performed at the BNL in the NSRL according to standardized procedures. For both ^1^H and ^56^Fe full-body IR mice were placed in individual polypropylene boxes with 4 mm holes drilled to produce a stress-free environment. LET levels for both particle radiations were held constant and the average dose-rate of 16.7 ± 5 cGy/min for ^1^H-IR and 5 ± 0.5 cGy/min for ^56^Fe-IR to deliver a cumulative dose of 90 cGy for ^1^H and 15 cGy for ^56^Fe, respectively. Constant energy of 1,000 MeV/nucleon (n) was used to deliver both, ^1^H- and ^56^Fe-IRs. Mice exposed to low-dose particle IR were driven back to GeneSys Research Institute (GRI) animal facility from BNL for housing and experimental analysis. Control Non-IR mice for each ion species was sham-IR; that is, mice were placed in the same individual polypropylene boxes, taken to the irradiation “cave,” and placed on beam line platform for the same duration of the time for each ion, but not irradiated.

### 2.3. Medium Transfer Experiments in BM-EPCs after ^1^H-IR and ^56^Fe-IR

We isolated BM-EPCs from mononuclear cell (MNC) fraction of total bone marrow isolated from tubular bones by flushing tibiae and femurs of ^1^H-IR, ^56^Fe-IR, and Non-IR mice using density gradient centrifugation. MNCs were then cultured on 22 × 22 mm square glass coverslips (Fisher Scientific, Pittsburg, PA) precoated with 0.2% gelatin (Sigma, St Louis, MO) in 6-well dishes (Corning Inc., Corning, NY). BM-EPCs were expanded* ex vivo* in selective EBM-2 growth medium supplemented with bullet kit growth factors (Lonza, Hopkinton, MA) until they attained ~70–80% confluence as described previously [15, 28, 42]. These BM-EPCs cultured in EBM-2 growth medium have been previously characterized for the following markers: *β*-gal (biological EC marker–cells were grown from Tie2/LacZ mice) and c-kit (stem/progenitor cell marker) wherein we demonstrated that 95–100% of cells by days 4 and 6 were double positive for both markers [28]. We also used two additional markers, Isolectin-B4 and Flk-1, which also showed similar results by day 6 in culture [28]. In our recent publication we further determined the purity of our BM-EPC cultures for other lineage specific hematopoietic cells, wherein we have performed immunofluorescent staining of these cells with antibodies for Gr1/Ly-6G (neutrophils), F4/80 (macrophages and blood monocytes), CD45R/B220 (B lymphocytes), CD3*ε* (T lymphocytes), and TER-119 (erythrocytes and erythroid precursors) [13]. These BM-EPCs have been shown to be negative for B220, Cd3*ε*, and TER-119 markers by day 5 in culture, with a negligible 1.17 ± 0.7% positivity for Gr1 marker and ~19% positivity for F4/80 marker [13].

**Figure 1:**
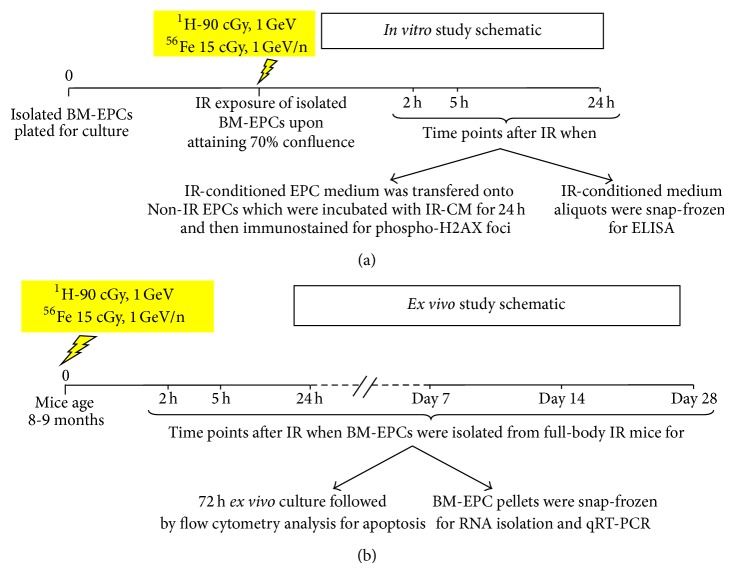
Diagrammatic representation of the experimental design to evaluate the effect of low-dose, whole body 90 cGy, 1 GeV ^1^H and 15 cGy, 1 GeV/n ^56^Fe-IR in BM-derived EPCs of 8–10 months old C57BL/6NTac. (a)* In vitro* study schematic for IR-conditioned medium transfer study to evaluate bystander responses in nonirradiated BM-EPCs over 24-hour time period after IR. (b)* Ex vivo* study schematic to evaluate the effects of full-body IR over 28 days on survival of BM-EPCs.

For IR-conditioned media transfer studies, two sets of BM-EPCs from the same WT mice that were IR with ^1^H or ^56^Fe and Non-IR controls were prepared as described previously [13, 43]. Upon attaining ~70% confluence one set of BM-EPCs was exposed to 90 cGy, 1 GeV of ^1^H-IR and 15 cGy, and 1 GeV/n of ^56^Fe-IR. After irradiations conditioned media (CM) from ^1^H- or ^56^Fe-IR-EPCs (^1^H- or ^56^Fe-IR-CM) and control Non-IR EPCs (Non-IR-CM) were collected at 2-, 5-, and 24-hour time points (Figure 1(a)). Prior to IR exposures, media were changed in all wells of both sets with fresh 3 mL of EBM-2 media without growth factors and incubated for 1 hour. The second set of Non-IR cells from the same mice was used as naïve (nonirradiated) EPCs for media transfer studies from their respective ^1^H-IR and ^56^Fe-IR exposed EPCs. ^1^H-IR- and ^56^Fe-IR-CM were filtered through a sterile 0.22 *μ*m membrane syringe filter and 2 mL of IR-CM collected at 2, 5 and 24 hours after IR was added onto corresponding mice Non-IR EPCs. Non-IR-CM were also collected, filtered, and transferred similarly. Naïve EPCs were incubated for 24 hours with ^1^H-, ^56^Fe-, and Non-IR conditioned media. EPCs from all three CM treatment conditions were collected at 2, 5, and 24 hours and processed for phosphorylated (p)-H2AX immunostaining as described below in Section 2.4. One mL of ^1^H-IR-, ^56^Fe-IR-, and Non-IR-CM was aliquoted and snap frozen in liquid nitrogen for protein analyses.

### 2.4. Immunofluorescent Staining

We assessed the formation and decay of p-H2AX foci in naïve EPCs treated for 24 hours with 2-, 5-, and 24-hour ^1^H-IR-, ^56^Fe-IR-, and Non-IR-CM EPCs. Cells on cover slips were washed with 1xPBS, fixed in 4% paraformaldehyde (PFA), and then incubated with primary anti-p-H2AX rabbit monoclonal antibody (Cat.9718S; Cell Signaling Technology, Danvers, MA). Alexa-488 goat anti-rabbit secondary antibody (Cat.A11008; Life Technologies, Grand Island, NY) was used to assay p-H2AX foci formation and decay over time in Non-IR-, ^1^H-IR-, and ^56^Fe-IR-CM-treated* ex vivo* expanded EPCs. Topro-3 was used to visualize nuclei (Cat.T3605; Life Technologies).

### 2.5. Confocal Microscopy and Analysis

Laser scanning confocal microscope (LSM 510 Meta, ZEISS, Thornwood, NY) was used to obtain immunofluorescent images at ×200 magnification. The analyses of p-H2AX foci were performed using a computer assisted image analysis algorithm based on pixel and color distribution. Data analysis was performed using stringent constraint of not including cells with apoptotic features or micronuclei for p-H2AX analysis. All time points after ^1^H and ^56^Fe-IR were plotted as percent cells with an N of p-H2AX foci compared to Non-IR controls and by quantifying cells with ≥2 p-H2AX foci/cell.

### 2.6. Enzyme-Linked Immunosorbent Assay (ELISA)

Conditioned media from BM-EPCs after ^1^H-IR, ^56^Fe-IR, and Non-IR were collected at 2, 5, and 24 hours after IR and processed for mouse multiplex cytokine ELISA using manufacturer protocol (Signosis, Santa Clara, CA). Following 9 cytokines, chemokines and growth factors were analyzed: interleukin-1 alpha (IL-1*α*), interleukin-1 beta (IL-1*β*), monocyte chemoattractant protein-1 (MCP-1), Rantes, microphage inflammatory protein-1 alpha (MIP-1*α*), granulocyte colony-stimulating factor (G-CSF), granulocyte macrophage colony-stimulating factor (GM-CSF), stem cell factor (SCF), and tumor necrosis factor-*α* (TNF-*α*). Absorbance readings at 450 nm were taken using Tecan Spectra model 96-well Microplate Reader (MTX Lab Systems, Vienna, VA) and data plotted using respective standard graphs obtained for each protein. Data analyzed was categorized into two separate groups: cytokines/chemokines and growth factors.

### 2.7. Apoptosis Assay of* Ex Vivo* Expanded BM-EPCs from ^1^H and ^56^Fe Full-Body Irradiated Mice over 28 Days

To assess the effects of ^1^H-IR and ^56^Fe-IR on survival of EPCs* ex vivo*, we isolated BM-EPCs from the total bone marrow of the full-body IR mice for short-term (2, 5, and 24 hours) and long-term (7, 14, and 28 days) time points after IR, as described in [15, 28, 42] and in Section 2.3 (Figure 1(b)). Isolated BM-EPCs from each ^1^H-IR and ^56^Fe-IR mice were cultured for 72 hours* ex vivo* in EPC selective EBM-2 media supplemented with bullet kit growth factors (Lonza), on 15 mm circular glass coverslips (Electron Microscopy Sciences, Hatfield, PA) precoated with 0.2% gelatin in 24-well dishes (Corning Inc.). At the end of 72 hours after initial seeding for both short- and long-term time points, BM-EPCs were trypsinized and harvested along with the supernatant growth media. No media change was done while BM-EPCs were in culture for 72 hours after seeding. Harvested cells were immunostained using Annexin V-FITC Apoptosis detection kit (eBiosciences Inc., San Diego, CA) as per manufacturer protocol and propidium iodide (final concentration 1 ug/mL). Cells were analyzed by flow cytometry analysis to evaluate ^1^H- or ^56^Fe-IR induced apoptosis in BM-EPCs. Annexin V was used to detect the cells in early stages of apoptosis and propidium iodide (PI) was used to identify necrotic cells. Data analyzed was plotted as percent (%) change in double Annexin V/PI (+) cells for full-body ^1^H-IR and ^56^Fe-IR* ex vivo* selected BM-EPCs compared to Non-IR BM-EPCs that were set at 100%.

### 2.8. Gene Expression Analysis and qRT-PCR

RNA from snap-frozen BM cells was isolated using RNeasy Mini Kit (QIAGEN, Valencia, CA). After isolation total RNA was converted to cDNA using the TaqMan Reverse Transcription Kit (Life technologies). qRT-PCR was performed on two genes (Bax and Bcl-2) that are known to play a significant role in the regulation of cell apoptosis. The samples were analyzed using Applied Biosystems 7300 Real Time PCR machine and software.

### 2.9. Statistical Analysis

All results were expressed as mean ± SEM and plots were obtained. Statistical analysis was performed on the data by one-way ANOVA (Stat View Software, SAS Institute Inc.; Gary, NC). Differences were considered significant at *P* < 0.05.

## 3. Results

### 3.1. Nonirradiated BM-Derived EPCs Treated with ^1^H-IR and ^56^Fe-IR Conditioned Media Exhibit Radiobiological Bystanders Responses* In Vitro*

**Figure 2:**
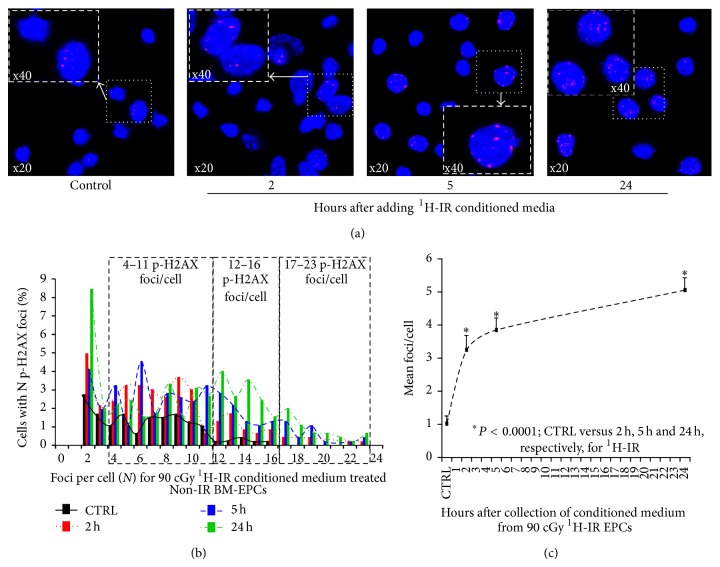
Nonirradiated BM-EPCs treated with ^1^H-IR conditioned media exhibit increased number of p-H2AX foci/cell* in vitro*. (a) Representative confocal images of p-H2AX 24 h after treatment of Non-IR BM-EPCs with ^1^H-IR-CM medium collected from duplicate set of respective BM-EPCs 2, 5, and 24 hours after 90 cGy ^1^H-IR. (b) Mean p-H2AX foci distribution (with ≥2 p-H2AX foci/cell) in BM-EPCs treated with Non-IR and ^1^H-IR conditioned media (CM). Foci distribution plot for % of Non-IR BM-EPCs with a given number (*N*) of foci after treatment for 24 h with CM from 90 cGy ^1^H-IR BM-EPCs at 2 h (red bars and dotted lines), 5 h (blue bars and dashed lines), and 24 h (green bars and dashed/dotted lines) compared to Non-IR controls (black bars and solid lines). For clarity of data presentation and due to no difference in the number of p-H2AX foci/cell between Non-IR and ^1^H-IR treatment groups, graphs represent distribution of p-H2AX foci/cell after excluding the cells with zero and 1 foci/cell. (c) Mean p-H2AX foci/cell plotted for control, 2 h, 5 h, and 24 h time points after treatment of Non-IR BM-EPC with ^1^H-IR CM. Graphs represent mean ± SEM of the pooled data from 5-6 independent biological samples/experiments. Statistical significance was assigned when *P* < 0.05.

We determined whether non-IR BM-EPCs may show evidence of bystander responses in media transfer experiment after treatment with ^1^H-IR and ^56^Fe-IR conditioned BM-EPCs media as described before [13]. There was a steady and significant increase in the mean p-H2AX foci/cell for Non-IR BM-EPCs treated with ^1^H-IR-CM. Compared to control CM-treated Non-IR BM-EPCs, there was 2–4-fold increase in the percent of cells with more than 4–11 p-H2AX foci/cell for ^1^H-IR-CM-treated cells (Figures 2(a) and 2(b)). There was less than 0.3% of cells with 12–16 p-H2AX foci/cell in control CM-treated BM-EPC; whereas 1.5–4% of ^1^H-IR CM-treated naïve BM-EPC had 12–16 p-H2AX foci/cell. Furthermore, BM-EPCs treatment for 24 hours with 2, 5, and 24 h ^1^H-IR-CM revealed 0.3–2% of cells with more than 17–23 p-H2AX foci/cell versus no cells with 17–23 p-H2AX foci/cell in control Non-IR-CM treated BM-EPCs (Figures 2(a) and 2(b)). These findings suggest that Non-IR BM-EPCs treated with ^1^H-IR-CM exhibit significant bystander responses up to 24 hours* in vitro*. We also determined the mean p-H2AX foci/cell induced in naïve BM-EPCs after 24-hour incubation with IR-CM at 2, 5, and 24-hour time point after IR. There was a steady and significant increase in mean p-H2AX foci/cell for naïve BM-EPCs treated with IR-CM from ^1^H-IR BM-EPCs at every time point compared to EPCs treated with Non-IR-CM, with a ~400% increase for 24 hour post-IR-CM treated naïve EPCs (Figure 2(c)).

**Figure 3:**
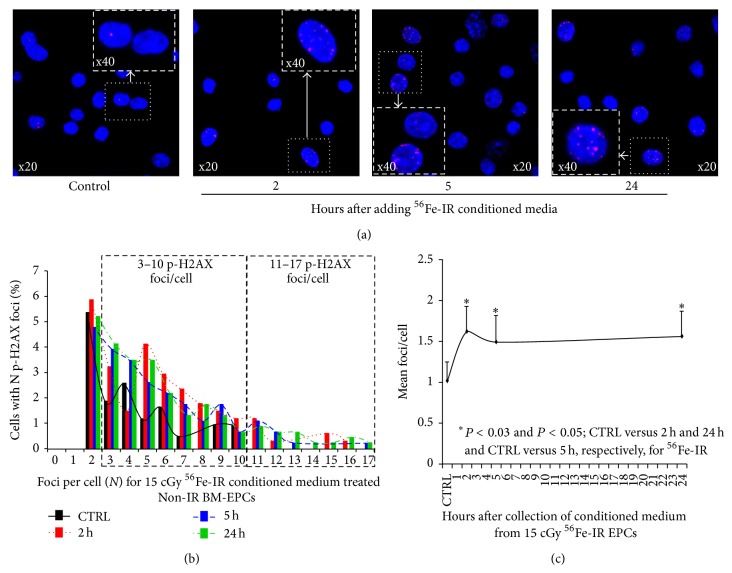
Nonirradiated BM-EPCs treated with ^56^Fe-IR conditioned media exhibit increased number of p-H2AX foci/cell* in vitro*. (a) Representative confocal images of p-H2AX 24 h after treatment of Non-IR BM-EPCs with ^56^Fe-IR-CM medium collected from duplicate set of respective EPCs 2, 5, and 24 h after 15 cGy ^56^Fe-IR. (b) Mean p-H2AX foci distribution (with ≥2 p-H2AX foci/cell) in BM-EPCs treated with Non-IR- and ^56^Fe-IR conditioned media (CM). Foci distribution plot for % of Non-IR BM-EPCs with a given number (*N*) of foci after treatment for 24 h with CM from 15 cGy ^56^Fe-IR BM-EPCs at 2 h (red bars and dotted lines), 5 h (blue bars and dashed lines), and 24 h (green bars and dashed/dotted lines) compared to Non-IR controls (black bars and solid lines). Due to no difference in the number of p-H2AX foci/cell between Non-IR and ^56^Fe-IR treatment groups, graph represents distribution of p-H2AX foci/cell after excluding the cells with zero and 1 foci/cell. (c) Mean p-H2AX foci/cell plotted for control, 2 h, 5 h, and 24 h treatment time point after treatment of BM-EPCs with ^56^Fe-IR CM. Graphs represent mean ± SEM of the pooled data from 5 to 6 independent biological samples/experiments. Statistical significance was assigned when *P* < 0.05.

Compared to control Non-IR CM-treated BM-EPCs, there was 2–4-fold increase in the percent of cells with more than 3–10 p-H2AX foci/cell for ^56^Fe-IR-CM-treated cells (Figures 3(a) and 3(b)). Furthermore, only ^56^Fe-IR CM-treated BM-EPCs revealed 0.3–1.3% of cells with more than 11–17 p-H2AX foci/cell at 2, 5, and 24 hours after treatment (Figures 3(a) and 3(b)). These findings suggest that Non-IR BM-EPCs treated ^56^Fe-IR-CM exhibit significant bystander responses up to 24 hours* in vitro*. We also determined the mean p-H2AX foci/cell induced in naïve BM-EPCs after 24-hour incubation with IR-CM at 2, 5, and 24-hour time point after ^56^Fe-IR. There was a significant increase in mean p-H2AX foci/cell for naïve BM-EPCs treated with ^56^Fe-IR-CM at every time point compared to EPCs treated with Non-IR-CM, with a ~160% increase for 2–24 hour post-IR-CM treated naïve EPCs (Figure 3(c)). It should be noted that the percent of mean pH2AX foci/cell in ^56^Fe-IR-CM treated naïve EPC was twice as low compared to ^1^H-IR-CM treated naïve EPCs. This finding could be directly attributed to significant cell death observed in ^56^Fe-IR-CM treated EPCs over 24 hours (data not shown).

### 3.2. Inflammatory Cytokines Are Significantly Increased in ^1^H-IR and ^56^Fe-IR Conditioned Medium

**Figure 4:**
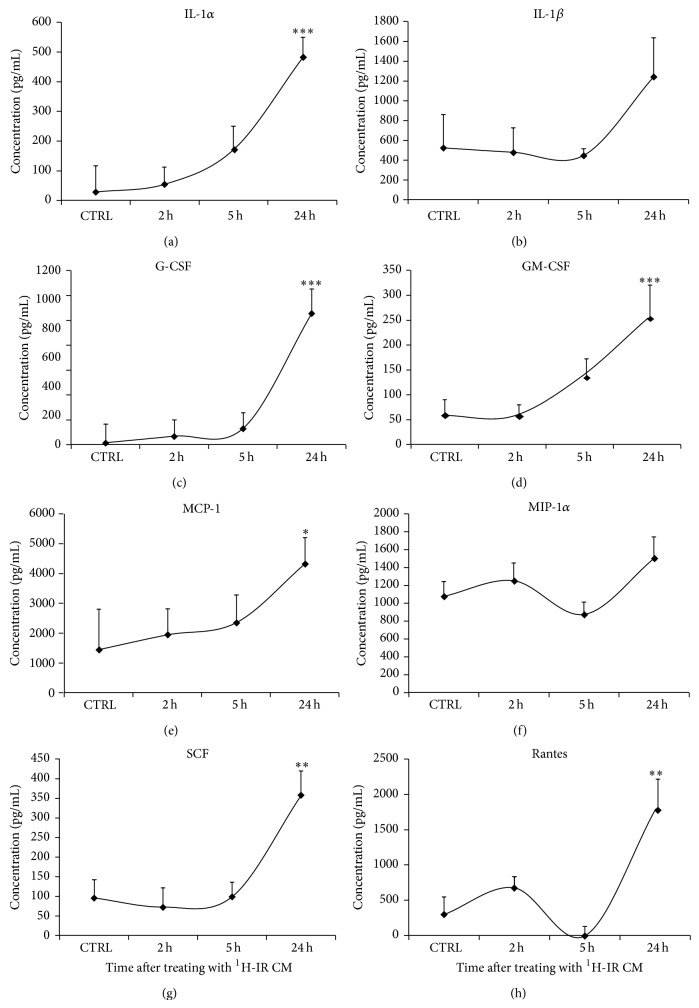
Cumulative levels of inflammatory cytokines and chemokines are significantly increased in ^1^H-IR conditioned medium. Graphic representation of IR-induced increases in the cumulative concentration (pg/mL) of cytokines, chemokines, and growth factors in CM from 90 cGy ^1^H-IR BM-EPCs* in vitro* at 2, 5, and 24 h after IR for (a) IL-1*α*, (b) IL-1*β*, (c) G-CSF, (d) GM-CSF, (e) MCP-1, (f) MIP-1*α*, (g) SCF, and (h) Rantes. Graphs represent mean ± SEM of the pooled data from 3 independent biological samples/experiments. Statistical significance was assigned when *P* < 0.05.

In 2006 Bubici et al., demonstrated that the convergence of IR-mediated effects results in inflammation due to increased levels of various cytokines and chemokines that generate reactive oxygen and nitrogen species [44]. We sought to determine the effect of ^1^H-IR on production and accumulation of cytokines, chemokines, and growth factors, such as IL-1*α*, IL-1*β*, MCP1, MIP-1*α*, Rantes, G-CSF, GM-CSF, and SCF in BM-EPCs, all of which are known to be elevated within minutes to hours after IR [4]. ELISA analysis of conditioned media from ^1^H-IR BM-EPCs showed a gradual increase in the levels of several cytokines, chemokines, and growth factor, when compared to Non-IR-CM. The maximum and statistically significant increases (2–53-fold) in IL-1*α*, MCP-1, Rantes, G-CSF, GM-CSF, and SCF were observed in the culture media of ^1^H-IR BM-EPCs at 24 hours (Figures 4(a), 4(c)–4(e), 4(g), and 4(h) and Table 1). Although, IL-1*β* and MIP-1*α* levels in ^1^H-IR BM-EPC culture media were slightly elevated (~39–136%) by 24 hours, it was not significant when compared to Non-IR EPC media (Figures 4(b) and 4(f) and Table 1). These findings suggest that in BM-EPCs, ^1^H-IR at 90 cGy induces accumulation of several cytokines and growth factors that have been directly implicated in mediating bystander responses in BM-derived EPCs [11, 13].

**Figure 5:**
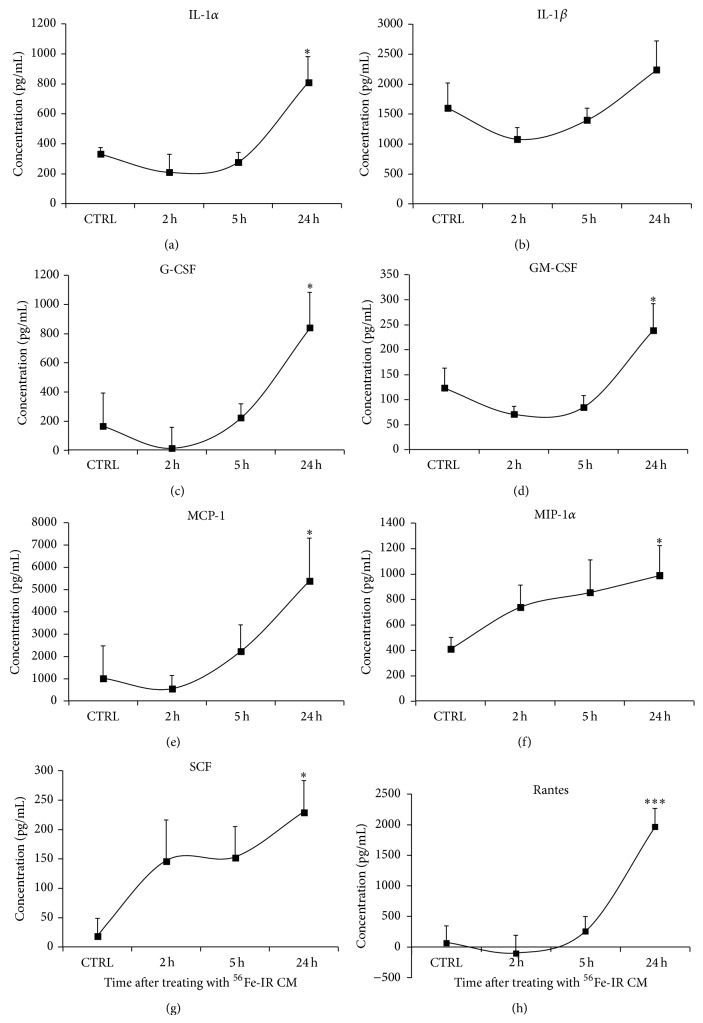
Cumulative levels of inflammatory cytokines and chemokines are significantly increased in ^56^Fe-IR conditioned medium. Graphic representation of IR-induced increase in the cumulative concentration (pg/mL) of cytokines, chemokines, and growth factors in CM from 15 cGy ^56^Fe-IR BM-EPCs* in vitro* at 2, 5 and 24 hours post-IR for (a) IL-1*α*, (b) IL-1*β*, (c) G-CSF, (d) GM-CSF, (e) MCP-1, (f) MIP-1*α*, (g) SCF, and (h) Rantes. Graphs represent mean ± SEM of the pooled data from 3 independent biological samples/experiments. Statistical significance was assigned when *P* < 0.05.

Similar to studies with ^1^H-IR BM-EPCs we also determined the accumulation of cytokines, chemokines, and growth factors in the media of BM-EPCs irradiated with 15 cGy, 1 GeV/n of ^56^Fe-IR. ELISA analysis of conditioned media from ^56^Fe-IR BM-EPCs showed a gradual increase in the levels of several cytokines, chemokines, and growth factor, when compared to Non-IR-CM. Maximum and statistically significant increase (1.4–22-fold) in IL-1*α*, MCP-1, MIP-1*α*, Rantes, G-CSF, GM-CSF, and SCF was observed by 24 hours (Figure 5(a) and 5(c)–5(h) and Table 2). Although, IL-1*β* level in ^56^Fe-IR EPC media were slightly elevated (~40%) by 24 hours, it was not significant when compared to Non-IR EPC media (Figure 5(b) and Table 2). These findings suggest that ^56^Fe-IR at 15 cGy induces accumulation of several cytokines and growth factors that have been directly implicated in mediating bystander responses [11, 13].

### 3.3. Full-Body ^1^H-IR and ^56^Fe-IR Induce Cyclical Increases in BM-EPC Apoptosis over 28 Days after IR

**Figure 6:**
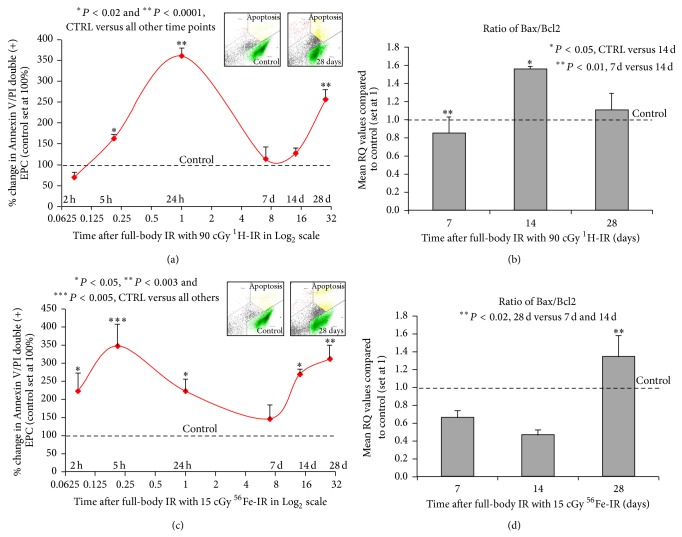
Full-body ^1^H-IR and ^56^Fe-IR induces early (2–24 h) and delayed (14–28 days) apoptosis in BM-EPCs* ex vivo*. Graphic representation of mean % change in Annexin V and propidium iodide (P.I) double positive (+) BM-EPCs cultured* ex vivo* for 72 h (solid red line) after full-body single-dose IR of (a) 90 cGy ^1^H-IR mice and (c) 15 cGy ^56^Fe-IR mice at 2, 5, and 24 hours and 7, 14, and 28 days after IR. The corresponding control for each time point was set at 100%. Insets in (a) and (c) are representative flow cytometry analysis plots for corresponding control and 28-day time points. Graphic representation of qRT-PCR analysis, mean RQ values compared to control (which was set at 1) of BM-EPCs from full-body single-dose IR of (b) 90 cGy ^1^H-IR mice and (d) 15 cGy ^56^Fe-IR mics at 7, 14, and 28 days after IR for ratio of Bax/Bcl2. The corresponding control for each time point was set at 1. Graphs represent mean ± SEM of the pooled data from 5-6 independent biological samples/experiments. Statistical significance was assigned when *P* < 0.05.

To determine the effect of full-body ^1^H-IR on* ex vivo* apoptosis, MNC isolated from total bone marrow were plated in 24-well plates at 2, 5, and 24 hours and 7, 14, and 28 days after ^1^H-IR. BM-EPC apoptosis was determined 72 hours after plating using flow cytometry analysis of BM-EPCs double stained with Annexin V and propidium iodide. Our results revealed that compared to control Non-IR BM-EPCs, in full-body ^1^H-IR EPCs cultured for 72 hours* ex vivo* there was 50% and 350% increases in BM-EPC apoptosis at 5 and 24 hours, respectively (Figure 6(a)). By day 7 the apoptosis was decreased to near control Non-IR levels. However, there was a second 250% increase in BM-EPC apoptosis in ^1^H-IR EPCs on day 28 (Figure 6(a)). This data indicates that there is a cyclical increase, early at 5 hours and delayed at 28 days, in BM-EPC apoptosis after a single full-body low-dose ^1^H-IR.

Accordingly, flow cytometry analysis of Annexin V/PI double positive cells revealed that 2, 5, and 24 hours after full-body ^56^Fe-IR there was 250–350% increase in BM-EPC apoptosis, with the peak 350% increases in apoptosis after ^56^Fe-IR at 5 hours (Figure 6(c)). By day 7 the apoptosis in ^56^Fe-IR BM-EPCs was decreased to near control Non-IR levels. However, there was a gradual increase in BM-EPC apoptosis in ^56^Fe-IR mice between days 14 and 28, with maximum 320% increase in apoptosis on day 28 (Figure 6(c)). This data indicates that there is a cyclical increase, early at 5 hours and delayed at 28 days, in BM-EPC apoptosis after a single full-body low-dose ^56^Fe-IR.

### 3.4. ^1^H-IR and ^56^Fe-IR Modifies Expression of Cell Cycle and Apoptosis Regulating Genes in BM-EPCs* Ex Vivo*

To determine whether full body ^1^H-IR may affect expression of Bax and Bcl-2 (two well-known regulators of survival and apoptosis) [45, 46], total RNA from ^1^H-IR BM-EPCs were processed for qRT-PCR. Because early effects of IR may be a nonspecific global shut-down of transcription and translation [47] we examined the gene expression in our samples at the later time points, that is, 7, 14, and 28 days after ^1^H-IR. Because the ratio of Bax protein, an inducer of apoptosis, to Bcl-2 protein, an inhibitor of apoptosis, could regulate survival or apoptosis after a stimulus, such as, ionizing radiation [48, 49], we evaluated the ratio of Bax/Bcl-2 expression. The ratio of Bax to Bcl-2 was decreased ~20% (*P* < 0.01) on day 7, which coincided with a significant decrease in BM-EPC apoptosis on day 7 compared to 24 h after ^1^H-IR (Figure 6(a)). There was ~60% (*P* < 0.05) increase in Bax/Bcl-2 ratio on day 14 after ^1^H-IR (Figure 6(b)) which coincided with the beginning of the increase in apoptosis in ^1^H-IR BM-EPCs between 14 and 28 days (Figure 6(a)). These results suggest, at least in part, the increase in the ratio of Bax/Bcl-2 expression may be responsible for induction of apoptosis in ^1^H-IR BM-EPCs.

As for ^1^H-IR BM-EPCs, we also examined the gene expression in ^56^Fe-IR BM-EPCs samples at 7, 14, and 28 days after ^56^Fe-IR. The ratio of Bax to Bcl-2 was decreased ~65% on day 7 (Figure 6(d)), which coincided with a significant decrease in BM-EPC apoptosis on day 7 compared to 5 and 24 h after ^56^Fe-IR (Figure 6(c)). There was a further ~15% decrease in Bax/Bcl-2 ratio on day 14 after ^56^Fe-IR (Figure 6(d)). However, compared to 7 and 14 days, there was more than 2-fold (*P* < 0.02) increase in the Bax/Bcl-2 ratio on day 28 (Figure 6(d)), which correlated with significant increase in apoptosis in ^56^Fe-IR BM-EPCs on day 28 after IR (Figure 6(c)). These results suggest at least in part the increase in ratio of Bax/Bcl-2 expression may be responsible for induction of apoptosis in ^56^Fe-IR BM-EPCs on day 28. However, increased apoptosis on day 14 may not be associated with the changes in the regulation of mitochondrial proteins, such as Bax and Bcl-2.

## 4. Discussion

A growing body of evidence indicates that in the heart and other organ-tissues vascular homeostasis does not exclusively rely on proliferation of local endothelial cells (ECs) but also involves BM-derived EPCs [50]. Indeed, studies have demonstrated that in patients with CV risk factors, the number and migratory ability of EPCs isolated from peripheral blood is reduced [51] and EPC function is impaired [52]. In addition, a strong inverse correlation was reported between the number of circulating EPCs, vascular function, and the subject's combined Framingham CV factor score [53]. Furthermore, measurements of flow-mediated brachial-artery reactivity also revealed a significant relation between endothelial function and the number of EPCs, supporting a role for EPCs in the maintenance of endothelial integrity [54].

It is established that EPCs mobilized from the bone marrow into circulation in response to injury or stress are aided by numerous chemokines and growth factors [55] that are known to be elevated within minutes to hours after IR [4, 56]. Proinflammatory cytokines such as TNF-*α*, IL-1*α*, and IL-6 have been well documented to be regulated as a direct effect of gamma (*γ*)-IR in murine hematopoietic cells [57] and human epithelial cells [58]. However, high levels of proinflammatory cytokines after IR exposure can cause profound negative effects and perpetuate further DNA damage through induction of reactive oxygen and nitrogen species, which then may lead to the increased oxidative stress [59–61]. It has been shown that EPCs express lower levels of basal and stress-induced intracellular reactive oxygen species (ROS) than primary ECs because EPCs express higher levels of catalase, manganese superoxide dismutase (MnSOD), and glutathione peroxidase-1 (GPx-1) [62, 63]. Hence, inhibition of catalase, MnSOD, and GPx-1 [64] may increase ROS levels in EPCs, which in turn impairs EPC survival and migration [62]. As ischemic/damaged tissue is characterized by high levels of inflammatory cytokines which activate ROS production [65], it has been proposed that high levels of ROS metabolizing enzymes in EPCs are essential to maintain their survival during tissue regeneration after injury. Conversely, these findings suggest that an imbalance in ROS can contribute to EPC dysfunction and that oxidative stress may impair neovascularization, thereby contributing to the pathogenesis and the progression of CV disease risks.

Current understanding of low-dose space and terrestrial radiation and its biological effects is that direct damage of DNA in the nucleus causes cell death and mutations [12]. However, in the past two decades there have been numerous studies which suggest that radiation can cause damage in nonirradiated cells through radiobiological bystander responses (RBR) [66, 67]. The term “bystander effect” was used to describe the ability of a cell, affected by radiation, to cause damage in other cells not directly traversed by the initial radiation [68]. It was suggested that generation of reactive oxygen and nitrogen species in IR tissues is mediated by increase in cytokines and chemokines [44, 69–71] and this could be one of the main mechanisms for persistent nontargeted, RBR after IR [66, 67]. The role of the bystander responses in BM-derived EPC after ionizing particle radiation remains largely unknown. The main goal of this study was to determine whether space-type ^1^H- and ^56^Fe-IR may induce RBR in BM-derived EPCs and evaluate the long term survival capacity of BM-EPCs after particle radiation.

We postulate that low-dose space IR-induced DNA damage responses in BM progenitor cell populations, including EPCs, may be of long duration and this may lead to significant decrease in the number of these cells, as well as long-term loss of EC function of BM-EPCs. This may then pose significant degenerative risk on physiologic homeostasis in the organs and tissue under conditions of normal aging and on repair and regeneration processes under pathologic conditions, such as injury or ischemia.

The acute phase of full-body low-dose IR induces apoptotic and immunological responses in the organ-tissues, including the heart [72], and is usually characterized by a neutrophil infiltration in affected area where macrophages are responsible for the phagocytic clearance of the apoptotic cells [73, 74]. It has been shown that phagocytosis of IR-induced apoptotic cells can activate macrophages, which subsequently induce an inflammatory response in the surrounding tissue [75] by releasing various cytokines, superoxide, and nitric oxide [76]. This can provide a potential feedback loop mechanism perpetuating inflammatory response leading to endothelial cell dysfunction in the heart and stem and progenitor cell populations in the BM milieu, as well as in other organs and tissues. Our* in vitro* findings of significant increase in the levels of several cytokines and chemokines (known to induce radiobiological bystander responses) [11, 13] in ^1^H- and ^56^Fe-IR BM-EPC conditioned media taken together with cyclical increase in BM-derived EPC apoptosis in* in vivo* studies may suggest a possible perpetuating mechanism of long-lasting IR-induced effects in the BM cell populations, including BM-EPCs. These findings in BM-EPCs can be corroborated with IR-induced inflammatory changes in ECs resulting in modification of homeostasis and endothelial dysfunction [77].

NASA Human Research Program (HRP) identified space radiation as one of the space flight risk factors to the cardiovascular system which is vastly unknown and limited to information collected days to weeks after space missions [78]. In addition to the possible biological effects of exposure to ^1^H and high charge and energy (HZE) ions (e.g., ^56^Fe), astronauts are also subjected to another critical physiological stressor, microgravity, which has been shown to produce untoward effects on the hematopoietic system and the BM microenvironment, leading to altered hematopoiesis-immunity [79–83] and cytokine production [84–87]. While most studies to-date examining DNA damage in the bone marrow environment as a result of exposure to IR have focused on direct effects on the stem and progenitor cell populations in BM milieu, recent studies [88, 89] have suggested that cytokines and signals within the BM tissue may play a key role in the ability of stem and progenitor cells to respond appropriately to IR-induced DNA damage. The effects of microgravity coupled with exposure to ionizing space radiation, both low-linear energy transfer (LET) proton (^1^H) and high-LET iron (^56^Fe), would eventually put astronauts in long-duration space missions at a higher risk of development of thrombotic diseases [90], manifestation of previously asymptomatic CV disease [78], immune dysfunction, and reduced vascular function and perfusion [91–94].

Epidemiologic data on IR-induced cardiovascular diseases from radiotherapy patients [95–97], nonoccupational exposure [43, 98, 99], and occupational exposure has demonstrated that cardiovascular (CV) morbidity may occur within months or years, and CV mortality may occur within decades, after initial IR exposure. Since EPCs are embedded in the microenvironment of bone marrow stroma which is considered as the most concentrated reservoir [100] and are mobilized to the circulation in response to activation of several mobilizing signaling pathways [55, 101], ionizing radiation induced dysfunction in BM-EPCs can ultimately result in degenerative CV risks. Since the transition from proinflammatory to more anti-inflammatory environment is crucial for proper tissue recovery it is of the utmost importance that cell proliferation and resistance to radiation induced cell death in bystander cells is enhanced [4, 102].

## 5. Summary

It is important to substantiate here that studies using particle radiation such as proton and iron are not only important for future successful space exploration it is also vital for civilian population, as by 2012 more than 120,000 cancer patients in 16 counties [103] have been treated using particle radiation therapy, primarily protons but also including carbon and other HZE ions, with similar centers being planned and constructed every year. Therefore our studies may also provide a foundation for the development of therapeutic measures to prevent CV morbidity and mortality due to cancer radiotherapy (conventional and/or the particle), as well as accidental and occupational IR exposures.

## 6. Conclusions

The presence of persistent IR-induced DNA damage in BM-EPCs along with increased apoptosis and possible impairment in DNA-repair characteristics of BM stem and progenitor cells may lead to BM-EPC dysfunction. This could then lead to the increase in CV degenerative risks in the form of cardiac fibrosis and eventually loss of cardiac function. We conclude that longitudinal studies using low-dose proton and heavy ion (HZE) radiation studies are warranted to determine IR-induced long-term CV risks.

## Figures and Tables

**Table 1:** Represents % change and statistical significance values in cumulative levels of cytokine, chemokine, and growth factors collected 24 h after treatment with ^1^H-IR-CM, for control versus day 1.

^1^H-IR-CM	Cytokines and chemokines				Growth factors			
	IL-1*α*	IL-1*β*	MCP-1	MIP-1*α*	Rantes	G-CSF	GM-CSF	SCF
CTRL versus 1 day % increase	1541%↑	136%↑	197%↑	39%↑	486%↑	5337%↑	324%↑	271%↑

CTRL versus 1 day *P* value	^***^ *P* < 0.0003		^*^ *P* < 0.05		^**^ *P* < 0.002	^***^ *P* < 0.0001	^***^ *P* < 0.007	^**^ *P* < 0.002

Asterisk corresponds to the respective plots for cumulative levels of inflammatory cytokines and chemokines in ^1^H-IR conditioned medium (Figure 4).

**Table 2:** Represents % change and statistical significance values in cumulative levels of cytokine, chemokine, and growth factors collected 24 h after treatment with ^56^Fe-IR-CM, for control versus day 1.

^56^Fe-IR-CM	Cytokines and chemokines				Growth factors			
	IL-1*α*	IL-1*β*	MCP-1	MIP-1*α*	Rantes	G-CSF	GM-CSF	SCF
CTRL versus 1 day % increase	141%↑	40%↑	413%↑	140%↑	2230%↑	402%↑	92%↑	1107%↑

CTRL versus 1 day *P* value	^*^ *P* < 0.02		^*^ *P* < 0.04	^*^ *P* < 0.04	^***^ *P* < 0.0001	^*^ *P* < 0.02	^*^ *P* < 0.04	^*^ *P* < 0.02

Asterisk corresponds to the respective plots for cumulative levels of inflammatory cytokines and chemokines in ^56^Fe-IR conditioned medium (Figure 5).
