# Corrigendum to “Particle Radiation-Induced Nontargeted Effects in Bone-Marrow-Derived Endothelial Progenitor Cells”

**DOI:** 10.1155/2016/7958361

**Published:** 2016-01-06

**Authors:** Sharath P. Sasi, Daniel Park, Sujatha Muralidharan, Justin Wage, Albert Kiladjian, Jillian Onufrak, Heiko Enderling, Xinhua Yan, David A. Goukassian

**Affiliations:** ^1^Cardiovascular Research Center, GeneSys Research Institute, Boston, MA 02135, USA; ^2^Whitaker Cardiovascular Institute, Boston University School of Medicine, Boston, MA 02118, USA; ^3^Department of Integrated Mathematical Oncology, H. Lee Moffitt Cancer Center and Research Institute, Tampa, FL 33612, USA; ^4^Tufts University School of Medicine, Boston, MA 02118, USA

In the paper titled “Particle Radiation-Induced Nontargeted Effects in Bone-Marrow-Derived Endothelial Progenitor Cells” [1], there was an error in “Acknowledgments,” which should be corrected as follows.
